# Trends in the Adoption of Robotic Surgery for Common Surgical Procedures

**DOI:** 10.1001/jamanetworkopen.2019.18911

**Published:** 2020-01-10

**Authors:** Kyle H. Sheetz, Jake Claflin, Justin B. Dimick

**Affiliations:** 1 Department of Surgery, University of Michigan, Ann Arbor; 2 Center for Healthcare Outcomes and Policy, University of Michigan School of Medicine, Ann Arbor; 3 currently a medical student at University of Michigan School of Medicine, Ann Arbor

## Abstract

**Importance:**

Increasing use of robotic surgery for common surgical procedures with limited evidence and unclear clinical benefit is raising concern. Analyses of population-based trends in practice and how hospitals’ acquisition of robotic surgical technologies is associated with their use are limited.

**Objective:**

To characterize trends in the use of robotic surgery for common surgical procedures.

**Design, Setting, and Participants:**

This cohort study used clinical registry data from Michigan from January 1, 2012, through June 30, 2018. Trends were characterized in the use of robotic surgery for common procedures for which traditional laparoscopic minimally invasive surgery was already considered a safe and effective approach for most surgeons when clinically feasible. A multigroup interrupted time series analysis was performed to determine how procedural approaches (open, laparoscopic, and robotic) change after hospitals launch a robotic surgery program. Data were analyzed from March 1 through April 19, 2019.

**Exposures:**

Initiation of robotic surgery.

**Main Outcomes and Measures:**

Procedure approach (ie, robotic, open, or laparoscopic).

**Results:**

The study cohort included 169 404 patients (mean [SD] age, 55.4 [16.9] years; 90 595 women [53.5%]) at 73 hospitals. The use of robotic surgery increased from 1.8% in 2012 to 15.1% in 2018 (8.4-fold increase; slope, 2.1% per year; 95% CI, 1.9%-2.3%). For certain procedures, the magnitude of the increase was greater; for example, for inguinal hernia repair, the use of robotic surgery increased from 0.7% to 28.8% (41.1-fold change; slope, 5.4% per year; 95% CI, 5.1%-5.7%). The use of robotic surgery increased 8.8% in the first 4 years after hospitals began performing robotic surgery (2.8% per year; 95% CI, 2.7%-2.9%). This trend was associated with a decrease in laparoscopic surgery from 53.2% to 51.3% (difference, −1.9%; 95% CI, −2.2% to −1.6%). Before adopting robotic surgery, hospitals’ use of laparoscopic surgery increased 1.3% per year. After adopting robotic surgery, the use of laparoscopic surgery declined 0.3% (difference in trends, −1.6%; 95% CI, −1.7% to −1.5%).

**Conclusions and Relevance:**

These results suggest that robotic surgery has continued to diffuse across a broad range of common surgical procedures. Hospitals that launched robotic surgery programs had a broad and immediate increase in the use of robotic surgery, which was associated with a decrease in traditional laparoscopic minimally invasive surgery.

## Key Points

**Question:**

Given concerns that robotic surgery is increasing for common surgical procedures with limited evidence and unclear clinical benefit, how is the use of robotic surgery changing over time?

**Findings:**

In this cohort study of 169 404 patients in 73 hospitals, the use of robotic surgery for all general surgery procedures increased from 1.8% to 15.1% from 2012 to 2018. Hospitals that launched robotic surgery programs had a broad and immediate increase in the use of robotic surgery, which was associated with a decrease in traditional laparoscopic minimally invasive surgery.

**Meaning:**

These findings highlight a need to continually monitor the adoption of robotic surgery to ensure that enthusiasm for new technology does not outpace the evidence needed to use it in the most effective clinical contexts.

## Teaser

This cohort study characterizes trends in the use of robotic surgery for common surgical procedures.

## Introduction

Robotic surgery continues to diffuse across an increasingly broad range of surgical procedures. However, concerns have been raised that robotic surgery is more costly^1,2^ and may be no more effective^3,4^ than other established operative approaches, such as traditional laparoscopic minimally invasive and open surgery. With respect to costs, for example, robotic surgery has been associated with episode costs as much as 25% higher compared with laparoscopic surgery. There are also concerns about the rapid growth of robotic surgery in areas with limited evidence to support its use and little theoretical benefit or clinical rationale (eg, inguinal hernia repair). The US Food and Drug Administration (FDA) recently issued a warning against the use of robotic surgery for the treatment of breast and cervical cancers.^5^ In their communication, they expressed concerns about the lack of epidemiologic data characterizing the use of robotic surgery in real-world practice settings. Current estimates are limited to single-center studies,^6,7,8^ device manufacturers’ financial statements,^9^ and claims data, which may be inaccurate owing to unreliable coding.^10,11^ We used population-based data from a manually abstracted statewide clinical registry to characterize contemporary trends in the adoption of robotic surgery across a range of general surgical procedures, which now represent the largest market for the technology in the United States.

## Methods

### Data Source and Study Population

This cohort study used data from the Michigan Surgical Quality Collaborative (MSQC), an Agency for Healthcare Research and Quality–recognized patient safety organization. The MSQC represents a voluntary partnership between 73 Michigan hospitals and Blue Cross/Blue Shield of Michigan that focuses on clinical quality improvement for surgical care. Hospitals participating in the MSQC perform more than 90% of all surgical procedures in Michigan. The MSQC maintains a clinical registry using a standardized data collection platform, validated case-sampling methods, and trained nurse data abstractors at each participating site. Data accuracy is maintained through rigorous training, internal data audits, and annual site visits by MSQC program staff. This data source allowed us to identify robotic procedures with greater precision and accuracy than is possible using claims data. This study was approved by the University of Michigan institutional review board, which deemed the study exempt from informed consent owing to use of secondary data. This study was designed and reported in adherence to the Strengthening the Reporting of Observational Studies in Epidemiology (STROBE) reporting guideline.

We used data from the complete MSQC clinical registry file to identify all inpatient and outpatient general surgical episodes from January 1, 2012, through June 30, 2018. Procedures were identified and categorized by *Current Procedural Terminology* codes. We focused on general surgical procedures, which represent the clinical domain with the largest growth in robotic surgery. These files include additional information on patient age, demographic characteristics, and comorbid conditions in addition to detailed procedural information (eg, operative approach and anesthesia type), postoperative complications, death, and resource use (readmissions and emergency department visits).

### Outcomes

Our primary outcome of interest was the surgical approach—robotic, laparoscopic, or open. The MSQC data were manually abstracted, and data on surgical approach were derived directly from the operative reports rather than procedural codes. Procedures were considered robotic if surgeons reported using the surgical robot in their operative report. Cases in which a robotic procedure was unexpectedly converted to another approach (eg, conversion to open procedure for bleeding) were characterized as robotic because this was the original approach chosen by the surgeon.

### Statistical Analysis

Data were analyzed from March 1 through April 19, 2019. The purpose of this analysis was to characterize trends in the use of surgical approaches over time for common general surgical procedures. We first reported raw proportions that were not adjusted for patient or hospital characteristics. We evaluated trends by calculating the fold change in each approach over time by dividing the proportional use of robotic surgery in 2018 by the proportional use in 2012. We also calculated the annual increase or decrease in the proportional use of each approach using linear regression. The coefficient for study years, modeled as a continuous variable, is reported as the annual trend. We then replicated the overall analysis stratified by specific procedures to determine whether overall trends were influenced by changes in practice for certain procedures.

To determine how hospitals change their practices after they begin performing robotic surgery, we performed a multigroup interrupted time series analysis. During the study period, 23 of the 73 MSQC participating hospitals (31.5%) began performing robotic surgery (32 hospitals were already performing robotic surgery at the time that MSQC began collecting data on this approach in 2012). We determined the date of the first robotic general surgery procedure within each of the hospitals that adopted robotic surgery during the study period. We then centered all hospitals on this date and evaluated the trends in the proportional use of each approach in the years before and after the hospital performed its first robotic operation. We used linear splines to model absolute levels and trends in the periods before and after introduction of robotic surgery. This analysis was designed to test the incremental association of adopting robotic surgery with trends in surgical practice but not to make assumptions about what would have happened had the hospital not begun performing robotic surgery. Our primary analysis was not adjusted for specific procedures, but we generated estimates for each procedure group in a sensitivity analysis. We estimated cluster-robust standard errors to account for repeated observations within hospitals. We performed all statistical analyses using Stata, version 14.2 statistical software (StataCorp LLC).

## Results

Characteristics for the 169 404 patients and 73 hospitals are included in Table 1. The mean (SD) age for all patients was 55.4 (16.9) years; 90 595 (53.5%) were women and 78 809 (46.5%) were men. Cholecystectomy was the most common operation (62 854 [37.1%]). Of the 73 hospitals included in the study, 31 (42.5%) had fewer than 200 beds and 11 (15.1%) had at least 500 beds. Sixty-two hospitals (84.9%) were teaching hospitals, and the mean (SD) total surgical volume was 12 068 (10 933) cases.

**Table 1.** Patient and Hospital Characteristics, 2012-2018

Characteristic	Data
**Patients (n = 169 404)**	
Age, mean (SD), y	55.4 (16.9)
Race, No. (%)	
White	140 951 (83.2)
Black	20 128 (11.9)
No. of comorbid conditions, mean (SD)	2.5 (1.2)
Surgical approach, No. (%)	
Robotic	13 500 (8.0)
Laparoscopic	85 326 (50.4)
Open	70 587 (41.7)
Most common procedures, No. (%)	
Cholecystectomy	62 854 (37.1)
Colectomy	26 695 (15.8)
Ventral hernia repair	26 376 (15.6)
Inguinal hernia repair	23 751 (14.0)
Reflux surgery	7021 (4.1)
Complex cancer resections	4285 (2.5)
Proctectomy	1897 (1.1)
**Hospitals (n = 73)**	
Bed size, No. (%)	
<200	31 (42.5)
200-349	20 (27.4)
350-499	11 (15.1)
≥500	11 (15.1)
Not-for-profit, No. (%)	64 (87.7)
Council of teaching hospitals, No. (%)	62 (84.9)
Nurse-to-patient ratio, mean (SD)	1.9 (0.7)
Total surgical volume, mean (SD), No./y	12 068 (10 933)
Inpatient surgical volume, mean (SD), No./y	4120 (4163)
Outpatient surgical volume, mean (SD), No./y	7947 (6974)

**Figure 1.**
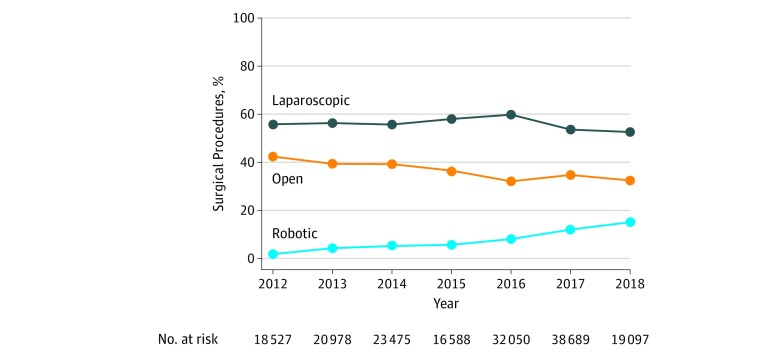
Temporal Trends in the Proportional Use of Robotic, Laparoscopic, and Open Surgery Data are from the Michigan Surgical Quality Collaborative from January 1, 2012, through June 30, 2018. These data reflect practices at all hospitals included in the study.

From January 2012 through June 2018, the use of robotic surgery for all general surgery procedures increased from 1.8% to 15.1% (8.4-fold change; slope, 2.1% per year; 95% CI, 1.9%-2.3%) (Figure 1 and Table 2). During the same period, the use of both laparoscopic and open surgery declined. For example, the proportional use of open surgery was 42.4% in 2012 compared with 32.4% in 2018 (0.8-fold change; slope, −1.5% per year; 95% CI, −1.8% to −1.2%) (eTable 1 and eTable 2 in the Supplement). Trends in robotic surgery use were similar for specific procedures, although for some, the magnitude of the increase was greater. For example, the use of robotic surgery for inguinal hernia repair increased from 0.7% to 28.8% from January 2012 through June 2018 (41.1-fold change; slope, 5.4% per year; 95% CI, 5.1%-5.7%).

**Table 2.** Trends in the Use of Robotic Surgery for Specific Procedures, 2012-2018

Procedure	Proportional Use, %		Fold Difference	Annual Slope (95% CI), %
	Year 2012	Year 2018		
All	1.8	15.1	8.4	2.1 (1.9-2.3)
Inguinal hernia repair	0.7	28.8	41.1	5.4 (5.1-5.7)
Ventral hernia repair	0.5	22.4	44.8	3.7 (3.5-3.9)
Colectomy	2.5	16.3	6.5	2.1 (1.8-2.4)
Reflux surgery	5.4	26.0	4.8	2.8 (2.3-3.2)
Proctectomy	3.1	26.7	8.6	4.0 (3.2-4.9)
Cholecystectomy	2.5	7.5	3.0	0.4 (0.3-0.5)
Complex cancer resections	2.1	3.9	1.9	0.4 (0.1-0.7)

**Figure 2.**
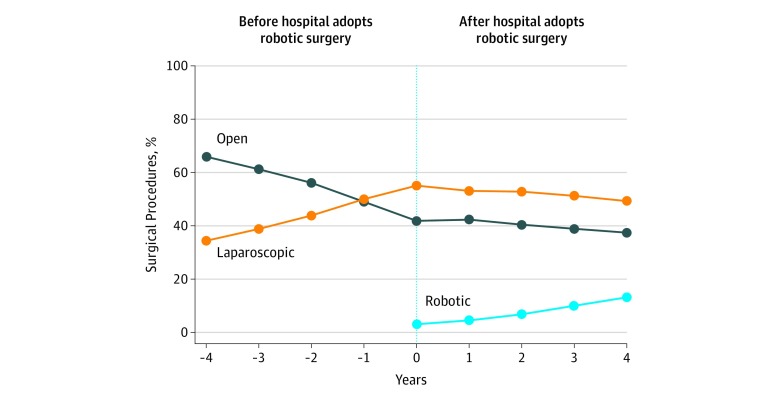
Changes in Procedure Approach After Hospitals Began Performing Robotic Surgery Proportional use of robotic, laparoscopic, and open approaches for general surgical procedures are shown in the 4 years before and after hospitals began performing robotic general surgery. From 2012 to 2018, 23 of 73 hospitals (31.5%) in the Michigan Surgical Quality Collaborative started performing robotic general surgery. These data are restricted to those hospitals.

The proportion of hospitals and surgeons performing robotic surgery increased from January 2012 through June 2018. For example, 8.7% of surgeons performed robotic general surgery in 2012 compared with 35.1% in 2018 (eFigure in the Supplement). During the study period, 23 hospitals (31.5%) began performing robotic surgery. In those hospitals, the use of robotic surgery increased from 3.1% in the first year to 13.1% in the fourth year after hospitals began performing robotic general surgery operations (overall mean in first 4 years, 8.8%; slope, 2.8% per year; difference, 2.8% [95% CI, 2.7%-2.9%]) (Figure 2 and Table 3). The use of laparoscopic surgery decreased from 53.2% to 51.3% after hospitals began performing robotic surgery (difference, −1.9%; 95% CI, −2.2% to −1.6%) (Table 3). Before hospitals performed robotic surgery, a trend toward greater use of laparoscopic surgery occurred (slope, 1.3% per year). A trend toward less laparoscopic surgery after hospitals began performing robotic surgery occurred (slope, −0.3% per year; difference, −1.6%; 95% CI, −1.7% to −1.5%). Results remained the same when stratified across specific procedures.

**Table 3.** Mean Use and Trends in Operative Approach Before and After Hospitals Began Performing Robotic Surgery for Specific Procedures

Procedure	Surgical Approach^a^								
	Laparoscopic			Open			Robotic		
	Before	After	Difference (95% CI)	Before	After	Difference (95% CI)	Before	After	Difference (95% CI)
All									
Proportional use, %	53.2	51.3	−1.9 (−2.2 to −1.6)	44.8	40.6	−4.2 (−4.5 to −3.9)	NA	8.8	8.8 (8.7 to 8.9)
Annual slope, %	1.3	−0.3	−1.6 (−1.7 to −1.5)	−1.6	−0.4	1.2 (1.1 to 1.3)	NA	2.8	2.8 (2.7 to 2.9)
Inguinal hernia repair									
Proportional use, %	12.8	17.5	4.7 (3.7 to 5.7)	74.0	60.4	−13.6 (−15.1 to −12.2)	NA	19.2	19.2 (18.7 to 19.7)
Annual slope, %	−1.0	0.5	1.5 (1.1 to 1.9)	−1.0	−1.1	−0.1 (−0.6 to 0.3)	NA	5.4	5.4 (5.1 to 5.6)
Ventral hernia repair									
Proportional use, %	27.4	26.7	−0.8 (−1.8 to 0.2)	73.0	64.2	−8.8 (−9.8 to −7.9)	NA	9.0	9.0 (8.6 to 9.3)
Annual slope, %	1.7	−0.2	−1.8 (−2.2 to −1.5)	−1.6	−0.8	0.8 (0.4 to 1.1)	NA	4.0	4.0 (3.8 to 4.2)
Colectomy									
Proportional use, %	32.7	37.7	5.0 (4.0 to 6.0)	64.1	52.5	−11.7 (−12.7 to −10.6)	NA	9.6	9.6 (9.2 to 10.0)
Annual slope, %	1.9	0.4	−1.5 (−1.8 to −1.1)	−2.2	−1.1	1.1 (0.8 to 1.5)	NA	3.1	3.1 (2.8 to 3.4)
Reflux surgery									
Proportional use, %	69.1	75.6	6.5 (4.2 to 8.9)	24.2	8.4	−15.8 (−18.3 to −13.3)	NA	14.2	14.2 (13.3 to 15.1)
Annual slope, %	−0.5	0.6	1.1 (0.6 to 1.6)	−0.4	−1.1	−0.7 (−1.0 to −0.4)	NA	3.8	3.8 (3.4 to 4.2)
Proctectomy									
Proportional use, %	16.9	15.0	−1.9 (−5.4 to 1.5)	79.3	66.8	−12.5 (−16.1 to −8.9)	NA	18.7	18.7 (16.9 to 20.5)
Annual slope, %	0.6	−0.2	−0.8 (−1.2 to −0.2)	−1.0	−1.2	−0.2 (−1.6 to 1.1)	NA	5.8	5.8 (4.8 to 6.9)
Cholecystectomy									
Proportional use, %	87.4	87.1	−0.2 (−1.0 to 0.5)	14.9	13.1	−1.7 (−4.4 to 1.0)	NA	5.9	5.9 (5.7 to 6.1)
Annual slope, %	−1.0	0.1	1.1 (0.9 to 1.2)	0.1	−0.2	−0.1 (−0.2 to 0.1)	NA	1.4	1.4 (1.3 to 1.5)
Complex cancer resections									
Proportional use, %	19.1	21.7	2.6 (−0.1 to 5.2)	78.2	74.7	−3.5 (−6.2 to −0.7)	NA	3.5	3.5 (2.9 to 4.1)
Annual slope, %	2.4	0.2	−2.2 (−3.2 to 1.1)	−2.8	−0.3	2.4 (1.4 to 3.6)	NA	0.6	0.6 (0.2 to 1.1)

Abbreviation: NA, not applicable.

^a^ Before and after indicate timing of adoption of robotic surgery.

## Discussion

This study used a unique, clinically oriented, and manually abstracted data source to characterize the use of robotic surgery across a wide range of common general surgical procedures. These data identify the correct procedure approach with greater precision and accuracy than claims. We found that the use of robotic surgery increased dramatically from 2012 to 2018. Although the use of robotic surgery increased across all procedures, for certain operations, such as inguinal hernia repair, practice patterns shifted by an order of magnitude toward greater use of robotics. We also found that the use of robotic surgery increased rapidly and diffused widely across numerous different procedures in the years after hospitals begin performing robotic surgery. This trend was associated with a decrease in the use of open and laparoscopic minimally invasive procedures, which for most surgeons was already considered a safe and effective approach when clinically feasible.

Recent work suggests that the United States now performs more robotic surgery than any other country in the world, although overall trends in other specialties (eg, urology) toward greater use of robotic surgery have been present for many years.^9^ Based on robotic device manufacturers’ financial statements, procedure volumes exceeded 600 000 in 2017, with the largest and fastest growing contributor being the field of general surgery.^9^ This finding suggests that the clinical footprint for robotic surgery will continue to increase as it has for more than a decade already. However, accurate data on how robotic surgery is being applied in contemporary practice is lacking. Prior studies are limited to single-center reports and claims-based analyses that may be inaccurate owing to unreliable coding.^6,7,8,10,11^ This inaccuracy is problematic because it may limit our ability to understand the clinical implications of this rapid change in practice. It also limits the ability of policy makers and regulators to scope oversight or, more broadly, the public health implications of rapid changes in surgical practice.

Within this context, regulators from the FDA recently expressed safety concerns about the rapidly growing use of robotic approaches for certain cancer operations.^5^ These concerns stem from the limited evidence of benefit (eg, fewer complications or better oncologic resection quality) for robotic surgery. For example, randomized clinical trials have failed to demonstrate the benefits of robotic surgery over other approaches in the treatment of rectal cancer^12^ and have shown even potentially worse outcomes in procedures for cervical cancer.^4^ Observational studies that compared robotic surgery with more established laparoscopic or open approaches have also failed to demonstrate superior outcomes after inguinal hernia repair,^8^ kidney resections,^1^ colectomy,^13,14,15,16^ or cholecystectomy.^7^ The discrepancy between the ongoing rapid adoption of robotic surgery and unclear clinical benefit highlights why accurate information on how it is being applied in contemporary surgical practice is necessary.

This study expands on prior work in several ways. We used manually abstracted data from a statewide surgical registry to ensure that our estimates reflect the true incidence of robotic surgery across a wide range of procedures, hospitals, and surgeons. Making further use of these unique data, we estimated how the initiation of robotic surgery within hospitals had broad associations with surgical practice for numerous procedures that differed in complexity, anatomical location, and surgical indications (eg, repair of a hernia vs removal of an organ). This investigation builds on existing literature, which has shown similar associations of an increase in robotic prostatectomy with hospital acquisitions of robotic systems.^17^ We also demonstrate that increasing use of robotic surgery changed existing trends toward greater use of laparoscopic surgery. For many common and low-risk procedures, such as cholecystectomy, conventional laparoscopic surgery is already the accepted standard of care. Laparoscopic approaches are also less expensive and can be performed by most general surgeons without robotics.^18^ This situation highlights a questionable trend: robotic surgery is replacing conventional laparoscopic approaches for procedures that may not be complex enough to warrant the consideration of an advanced, expensive, and unproven minimally invasive platform.

This study suggests that regulators and the surgical community can take additional steps to monitor the ongoing diffusion of robotic surgery and ensure that this trend does not lead to diminished patient safety. Because accurate data are necessary to inform the creation of appropriate safeguards, the FDA and the Centers for Medicare & Medicaid Services should consider providing coverage for robotic surgery with provisions for evidence development.^19^ This process has been previously used by the Centers for Medicare & Medicaid Services to create registries of patients treated with new and unproven surgical technologies (eg, carotid artery stenting). Use of these provisions would facilitate greater understanding of how robotic procedures are being used in real-world practice. Akin to postmarket surveillance of pharmaceuticals, such provisions would also create a common data resource from which the comparative safety and effectiveness of robotic operations can be evaluated by numerous investigators.

This action would also allow hospitals, which provide credentials to perform robotic surgery, to better understand where sufficient evidence suggests plausible benefit. At present, surgeons are largely able to use robotic surgery for any procedure at their professional discretion. As has been shown in the FDA warning and through prior studies, this discretionary use may place patients at risk for poor outcomes.^3^ Facilitating transparency around the allocation of robotic surgery would allow patients to make better collaborative decisions with their surgeons. After all, for many of the procedures we report in this study, little to no evidence suggests that robotic surgery increases patient safety or treatment effectiveness compared with other approaches.

### Limitations

Our results should be interpreted within the context of several limitations. Our clinical registry only captures data from Michigan and therefore may not be generalizable to the country as a whole. However, the MSQC represents a heterogeneous group of hospitals, surgeons, and practice settings. Furthermore, we report on the most common general surgery procedures performed in the United States. Changes in patient factors, such as obesity, may influence trends in procedure choice. Our estimates may therefore be limited by a lack of adjustment for patient characteristics. That said, adjusting for patient factors may introduce its own biases because no clinical consensus exists around how robotic procedures should be allocated. Much of this decision-making is based on case-by-case surgeon assessments and clinical nuance not captured in any registry. Our results are consistent across multiple different procedures, which also suggests that these trends are independent of unique clinical domains or disease processes. Our study is unable to account for how other nonclinical factors, such as marketing, may influence the adoption of robotic surgery. However, others have found that the chances of receiving robotic surgery were 2- to 5-fold greater in highly competitive vs noncompetitive health care markets.^20^ Moreover, evidence suggests that hospitals immediately begin advertising their acquisition of robotic surgical services through web-based and conventional health system marketing campaigns.^21^ These data are complementary to ours and suggest that the greatest forces driving robotic surgery adoption may be the technological imperative and economic pressures experienced by hospitals in certain health care markets.

## Conclusions

This study found that robotic surgery is rapidly diffusing across a broad range of common general surgical procedures. Trends toward greater use of the robotic platform appeared to occur rapidly after hospitals begin performing robotic surgery and were associated with a decrease in the use of established minimally invasive techniques, such as laparoscopic surgery. This trend was consistent across numerous specific procedures, even those for which conventional laparoscopic surgery is already considered standard of care and for which robotic surgery offers little theoretical clinical benefit to the patient. These findings highlight a need to continually monitor the diffusion of robotic surgery to ensure that enthusiasm for a new technology does not outpace the evidence needed to use it in the most effective clinical contexts.

## Notes

Supplement. **eTable 1.** Trends in the Use of Open Surgery for Specific Procedures, 2012-2018 **eTable 2.** Trends in the Use of Laparoscopic Surgery for Specific Procedures, 2012-2018 **eFigure.** Proportion of Hospitals and Surgeons Performing Any Robotic General Surgery in Michigan, 2012-2018
