# Supplementary material for: Trends in the Adoption of Robotic Surgery for Common Surgical Procedures
Source: JAMA Netw Open. 2020 Jan 10;3(1):e1918911. doi: 10.1001/jamanetworkopen.2019.18911 (PMC6991252; doi:10.1001/jamanetworkopen.2019.18911)
Supplement: Supplement. — eTable 1. Trends in the Use of Open Surgery for Specific Procedures, 2012-2018 eTable 2. Trends in the Use of Laparoscopic Surgery for Specific Procedures, 2012-2018 eFigure. Proportion of Hospitals and Surgeons Performing Any Robotic General Surgery in Michigan, 2012-2018 [file jamanetwopen-3-e1918911-s001.pdf]

Supplementary Online Content

Sheetz KH, Claflin J, Dimick JB. Trends in the adoption of robotic surgery for common surgical procedures. *JAMA Netw Open.* 2020;3(1):e1918911. doi:10.1001/jamanetworkopen.2019.18911

eTable 1. Trends in the Use of Open Surgery for Specific Procedures, 2012-2018
eTable 2. Trends in the Use of Laparoscopic Surgery for Specific Procedures, 2012-2018
eFigure. Proportion of Hospitals and Surgeons Performing Any Robotic General Surgery in Michigan, 2012-2018

This supplementary material has been provided by the authors to give readers additional information about their work.

© 2020 Sheetz KH et al. *JAMA Network Open*

**eTable 1**. Trends in the Use of Open Surgery for Specific Procedures, 2012-2018				
	**Proportional Use, %**			
Procedure	Year 2012	Year 2018	Fold Difference	Annual Trend, % per Year (95% CI)
**All**	42.4	32.4	0.8	-1.5 (-1.8 to -1.2)
**Inguinal hernia repair**	98.2	53.1	0.5	-8.1 (-8.4 to -7.7)
**Ventral hernia repair**	83.4	54.1	0.6	-3.8 (-4.1 to -3.6)
**Colectomy**	77.0	45.7	0.6	-4.1 (-4.4 to -3.8)
**Reflux surgery**	40.2	5.2	0.1	-3.6 (-4.0 to -3.3)
**Proctectomy**	87.9	63.8	0.7	-3.3 (-4.4 to -2.3)
**Cholecystectomy**	2.5	1.3	0.5	-3.6 (-3.7 to -3.5)
**Complex cancer resections**	86.1	72.7	0.8	-1.8 (-2.4 to -1.1)

**eTable 2**. Trends in the Use of Laparoscopic Surgery for Specific Procedures, 2012-2018				
	**Proportional Use, %**			
Procedure	Year 2012	Year 2018	Fold Difference	Annual Trend, % per Year (95% CI)
**All**	55.8	52.6	0.9	-0.5 (-0.7 to -0.3)
**Inguinal hernia repair**	0.8	18.0	22.5	2.6 (2.3 to 2.9)
**Ventral hernia repair**	16.6	23.5	1.4	0.2 (-0.1 to 0.4)
**Colectomy**	20.5	38.0	1.9	1.9 (1.6 to 2.2)
**Reflux surgery**	54.4	68.8	1.3	0.9 (0.3 to 1.4)
**Proctectomy**	9.0	9.5	1.1	-0.6 (-1.4 to 0.2)
**Cholecystectomy**	91.2	92.1	1.0	3.2 (3.0 to 3.4)
**Complex cancer resections**	11.8	23.3	2.0	1.4 (0.8 to 2.0)

© 2020 Sheetz KH et al. *JAMA Network Open*

eFigure. Proportion of Hospitals and Surgeons Performing Any Robotic General Surgery in Michigan, 2012-2018

Appendix Figure 1

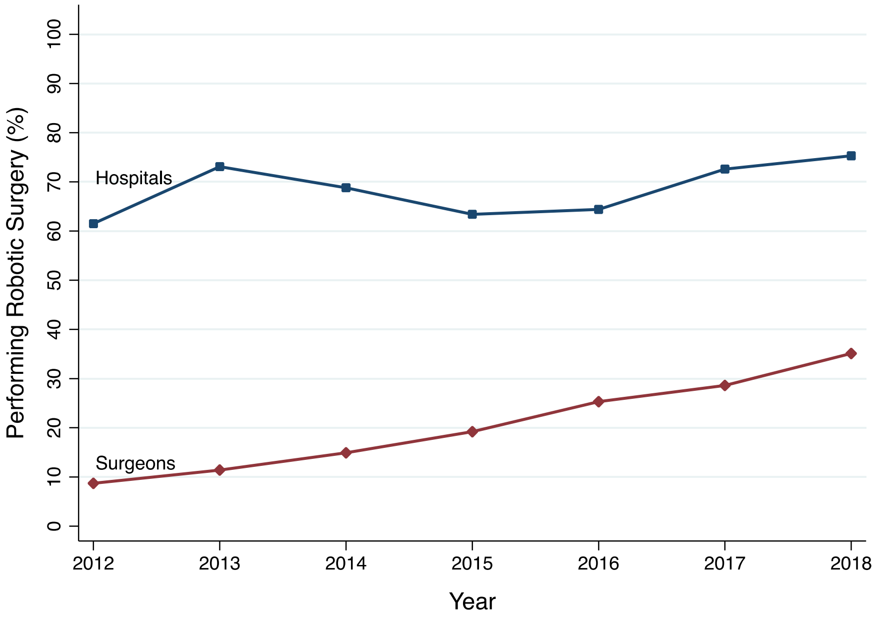

No. of Hospitals	52	52	64	71	73	73	73
No. of Surgeons	759	841	871	902	974	972	828

© 2020 Sheetz KH et al. *JAMA Network Open*
